# Polymyxin B nonapeptide potentiates the eradication of Gram-negative bacterial persisters

**DOI:** 10.1128/spectrum.03687-23

**Published:** 2024-02-23

**Authors:** Sun Ju Kim, Jeongwoo Jo, Jihyeon Kim, Kwan Soo Ko, Wonsik Lee

**Affiliations:** 1 School of Pharmacy, Sungkyunkwan University, Suwon, Republic of Korea; 2 Department of Microbiology, School of Medicine, Sungkyunkwan University, Suwon, Republic of Korea; JMI Laboratories, North Liberty, Iowa, USA

**Keywords:** persister, polymyxin B nonapeptide, colistin, Gram-negative bacteria, combination treatment

## Abstract

Antibiotic-resistant Gram-negative bacteria remain a globally leading cause of bacterial infection-associated mortality, and it is imperative to identify novel therapeutic strategies. Recently, the advantage of using antibacterials selective against Gram-negative bacteria has been demonstrated with polymyxins that specifically target the lipopolysaccharides of Gram-negative bacteria. However, the severe cytotoxicity of polymyxins limits their clinical use. Here, we demonstrate that polymyxin B nonapeptide (PMBN), a polymyxin B derivative without the terminal amino acyl residue, can significantly enhance the effectiveness of commonly used antibiotics against only Gram-negative bacteria and their persister cells. We show that although PMBN itself does not exhibit antibacterial activity or cytotoxicity well above the 100-fold minimum inhibitory concentration of polymyxin B, PMBN can increase the potency of co-treated antibiotics. We also demonstrate that using PMBN in combination with other antibiotics significantly reduces the frequency of resistant mutant formation. Together, this work provides evidence of the utilities of PMBN as a novel potentiator for antibiotics against Gram-negative bacteria and insights for the eradication of bacterial persister cells during antibiotic treatment.

**IMPORTANCE:**

The significance of our study lies in addressing the problem of antibiotic-resistant Gram-negative bacteria, which continue to be a global cause of mortality associated with bacterial infections. Therefore, identifying innovative therapeutic approaches is an urgent need. Recent research has highlighted the potential of selective antibacterials like polymyxins, which specifically target the lipopolysaccharides of Gram-negative bacteria. However, the clinical use of polymyxins is limited by their severe cytotoxicity. This study unveils the effectiveness of polymyxin B nonapeptide (PMBN) in significantly enhancing the eradication of persister cells in Gram-negative bacteria. Although PMBN itself does not exhibit antibacterial activity or cytotoxicity, it remarkably reduces persister cells during the treatment of antibiotics. Moreover, combining PMBN with other antibiotics reduces the emergence of resistant mutants. Our research emphasizes the utility of PMBN as a novel potentiator to decrease persister cells during antibiotic treatments for Gram-negative bacteria.

## INTRODUCTION

Antibiotic-resistant bacterial infections have become increasingly common during the past two decades to become one of the most significant burdens on public health (1). Today, among the bacterial pathogens, multidrug-resistant (MDR) Gram-negative bacteria pose a particular problem because of their encapsulated outer membrane (2). The negatively charged lipopolysaccharides (LPS) are stabilized by divalent cations such as Mg^2+^, and this creates a layer around the cell that hampers many antibacterial compounds from penetrating the envelope of Gram-negative bacteria (3). Also, the outer membrane hosts various efflux pumps, and these pumps export toxic compounds including antibiotics from inside the cell. The high frequency of MDR of Gram-negative bacteria is attributed to such properties of their outer membrane, which in the clinic, can result in an elevated risk of morbidity and mortality of patients (4). However, the treatment options for Gram-negative bacterial infections are becoming limited while MDRs are still elusively evolving into pan-drug resistant strains. Moreover, persister cells, a small subpopulation of these bacteria, show metabolically dormant and strong tolerance to most antibiotics. The tolerance to antibiotics in bacteria is achieved through the regulation of gene expression, while antibiotic resistance is acquired by mutations or genes related to antibiotic degradation. Since the persister population is highly tolerant to antibiotics, these cells cannot be removed from the culture by using antibiotics, even well above minimum inhibitory concentration (MIC). Also, recent studies have suggested that the persister population has been considered one of the major causes of treatment failure and the recurrence of infection (5). Therefore, antibacterials with a mechanism targeting the LPS or novel strategic combinations of known compounds are urgently needed.

Polymyxins, which are cationic and branched cyclic decapeptides, were discovered in the 1940s, and they are still used to treat multi-drug resistant Gram-negative bacteria including *Acinetobacter baumannii*, *Klebsiella pneumoniae*, and *Escherichia coli*. However, their use in the clinic is limited due to severe adverse effects such as neurotoxicity (6), and their potency against other clinically important Gram-negative bacteria is not outstanding (7). The latter is due to the rapid acquisition of resistance, formation of persister cells, or dissemination of resistance genes such as *mcr-1* by horizontal gene transfer (8). In particular, the dissemination of resistance genes raises further concerns since the co-occurrence of other resistance genes such as *bla_NDM_* and *bla_KPC_* that result in strong resistance to cephalosporins and carbapenems has recently been reported (9).

Co-treatment of polymyxins with other antibiotics such as carbapenems has been suggested to treat MDR infections including urinary tract bacterial infections (10). However, in the clinic, co-treatment of polymyxins with other antibiotics is still limited due to high nephrotoxicity. Alternatively, polymyxin derivatives that show a much lower cytotoxicity against human cells at concentrations well above the MIC (150×) of polymyxins while still recognizing the outer membrane of Gram-negative bacteria, were introduced (11). Among these, polymyxin B nonapeptide (PMBN), a polymyxin B derivative without the terminal amino acyl residue of polymyxin B, leads to elevated permeability of the outer membrane of Gram-negative bacteria without showing antibacterial activity by itself (12). Therefore, this mechanism of action of PMBN can potentiate other antibiotics that have cellular targets by facilitating their penetration through the cell membrane.

Here, we demonstrated that co-treatment of PMBN with other antibiotics, or PMBN alone, resulted in a significant reduction of the persister subpopulation. Furthermore, we showed that co-treatment with PMBN led to a decrease in the frequency of resistance acquisition against the co-treated antibiotics. This work demonstrates the utility of a co-treatment strategy that exploits a non-toxic derivative of polymyxins to potentiate antibiotics used in the clinic. This approach should be applicable to other cell membrane-targeting compounds to improve therapeutic potency against bacterial infections.

## MATERIALS AND METHODS

### Bacterial strains

A total of four bacterial species, *Acinetobacter baumannii*, *Klebsiella pneumoniae*, *Escherichia coli*, *Staphylococcus aureus,* and *Bacillus subtilis* were included. For *A. baumannii,* 07AC-032 and C010 were used; *K. pneumoniae,* ATCC 10031, ATCC 43816, SMC1204-109; *E. coli,* ATCC 25922 and MG1655; *S. aureus,* HG003; *B. subtilis*, BY79. *K. pneumoniae* ATCC 43816 and SMC1204-109 belong to the K2 serotype and both show hypermucoviscosity.

### Antimicrobial susceptibility test

Amikacin (AMK), ciprofloxacin (CIP), colistin (CST), cefotaxime (CTX), meropenem (MRP), polymyxin B nonapeptide (PMBN), and tetracycline (TET) were purchased from Sigma-Aldrich (St. Louis, MO). The testing was performed following the Clinical and Laboratory Standards Institute (CLSI) guideline and resistance was determined according to the breakpoint of CLSI M100-ED32 (13).

### Fluorescence inverted microscopy

Microscopy was performed on an agarose gel pad at room temperature using a Leica system (Leica DMi8 M; inverted microscope with 100× oil immersion objective lens). Images and movies were taken every hour. Overnight cultures of *E. coli* MG1655 and *S. aureus* HG003/pLOW-*ftsZ*-GFP were adjusted to OD_600_ = 0.5 and reinoculated at 1:100 ratio into trypticase soy broth containing 0.03 µg/L of meropenem and 1 mg/L PMBN as indicated (14). The cultures were then incubated at 37°C with shaking at 220 rpm. Samples were prepared and subjected to microscopy.

### Persister assay

Preliminarily, the MIC of isolates was measured and biphasic time-kill curves under antibiotic stress were verified by sampling for every 2 hours until 8 hours with initial inoculum size OD_600_ = 0.5 (15–21). For the persister assay, overnight cultures of all strains were adjusted to OD_600_ = 0.5 and inoculated at a 1:100 ratio into 15 mL LB media. They were grown until the optical density at 600 nm reached 0.5, then diluted to OD_600_ = 0.1. Each 15 mL culture was then treated with the antibacterial agents to induce persistent cells in a 50 mL conical tube. After 6 hours of antibiotic challenge at 37°C, 220 rpm, 1 mL of each culture was sampled and washed twice with phosphate-buffered saline (PBS) to eliminate the remaining antibiotics. The washed cells were serially diluted ten-fold in PBS and 5 µL of each bacterial dilution was spotted on LB agar plates. The surviving bacteria subpopulations, persisters, were indicated as CFUs/mL. To confirm whether the surviving colonies were persister cells, all colonies were collected from the spot assay plates and subjected to an antibiotic susceptibility assay to determine the MICs of antibiotics (22).

### Determination of eradication of persister cells by combinatorial treatment (PMBN and antibiotics) and comparison of the effects of PMBN and colistin on the combinatorial treatment

Bacterial cells in the exponential phase were diluted into 15 mL of LB media to OD_600_ = 0.1 and treated with antibiotics for 6 hours to induce persistence, as described above. Each of samples was washed with PBS twice and resuspended into 15 mL LB media after the treatment. The 2 mL of each suspension was prepared in 14 mL round bottom tubes. The cultures then were treated as follows: untreated, antibacterial agent only, PMBN at 1 and 2 mg/L, antibacterial agent with potentiator at 1 or 2 mg/L. After 4 hours of treatment, the CFUs/mL from each treatment were measured.

### Induction of spontaneous resistant mutants in the combinatorial treatment

To study the effects of PMBN on resistance acquisition, *E. coli* MG1655 was challenged with antibiotics in the presence or absence of 2 mg/L PMBN. Briefly, an overnight culture of MG1655 was adjusted to OD_600_ = 0.5, inoculated at a 1:100 ratio in 2 mL of Mueller-Hinton (MH) broth with or without 2 mg/L of PMBN and challenged overnight at 37°C, 220 rpm (23–27). The concentration of antibiotics for the first exposure, one-eighth of the MIC, was selected when the overnight cultures were grown to over OD_600_ 1.0 by a single-exposure to antibiotics. The sequential passaging was repeated, and MICs were determined at each passage.

### Statistical analysis

All experiments were carried out three times with biological replicates. The unpaired Student’s T test was used for comparison of two groups when available. The asterisks were used to indicate the statistical significance. One to three asterisks are corresponding to *P*-value at 0.05, 0.01, and 0.005.

## RESULTS AND DISCUSSION

### PMBN potentiates antibiotics against Gram-negative bacteria

**Fig 1:**
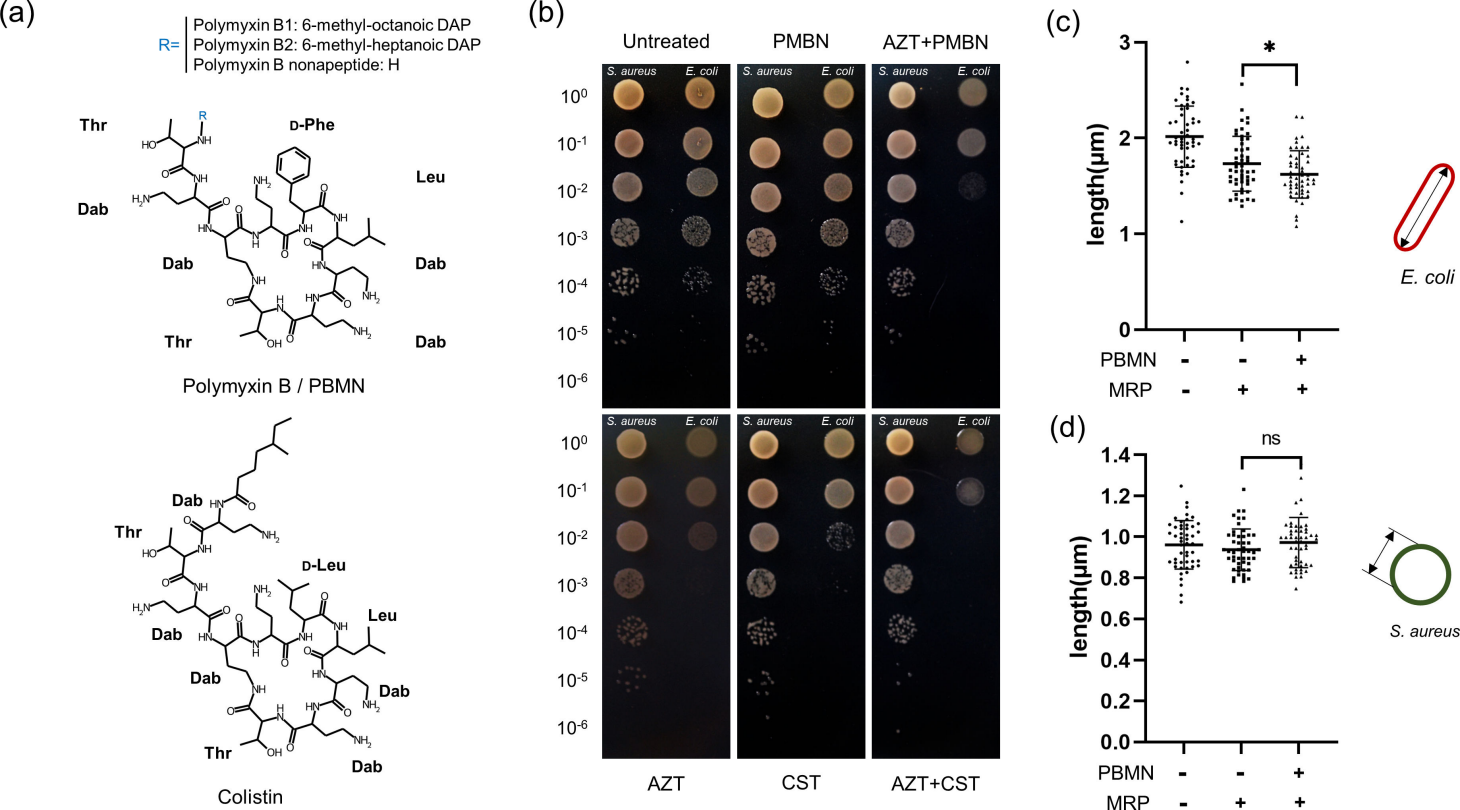
Polymyxin B nonapeptide (PMBN) potentiates antibiotics at sub-MIC levels. (a) PMBN lacks 6-methyl-octanoic/heptanoic DAP, resulting in decreased cytotoxicity. (b) Bacterial cells were challenged with aztreonam, and PMBN or colistin was added as a potentiator. *E. coli* MG1655 is in the right column, and *S. aureus* is in the left column in each spot dilution. PMBN itself does not show antimicrobial activity. Addition of PMBN sensitized Gram-negative bacteria 10^3^-fold. The sensitizing activity of PMBN is comparable to that of colistin. (**c, **d) *E. coli* MG1655 (c) and *S. aureus* (d) were treated with meropenem (MRP) only or in the presence of 1 mg/L PMBN for three hours. The cell width was measured using ImageJ (version 1.54d). Each fifty cells for *E. coli* and *S. aureus* were quantified. MRP only versus combinatory treatment is statistically significant at *P* = 0.029.

Selective killing strategies where only Gram-positive bacteria survive could have advantages in the clinic because of less perturbance of the native microbiota of the host (28). To seek desirable selective conditions with an agent less toxic to the host cell, we decided to modify CAP blood agar containing colistin and aztreonam which is widely used to selectively enrich Gram-negative bacteria. We replaced 10 mg/L colistin of CAP with 1 mg/L PMBN since PMBN itself has no antibacterial activity (MIC >64 mg/L) (Fig. S1). As shown in Fig. 1b, when we replaced 10 mg/L colistin with 1 mg/L PMBN in the presence of aztreonam, PMBN showed a similar increase in its potentiation compared to colistin while PMBN in the absence of aztreonam showed no antibacterial effect. Importantly, we found that the co-treatment of aztreonam with PMBN led to a higher synergism compared to that of colistin, since PMBN itself has no detectable antibacterial activity against *E. coli* at 100 mg/L (Fig. S1). As previously described, the potency of cell wall-targeting antibiotics such as beta-lactams is particularly increased in the presence of PMBN, and this may be due to the location of their cellular targets (29). The synergism and selectivity of co-treatment were validated in co-culture conditions of *E. coli* and *S. aureus* using meropenem (MRP), which exhibits broad-spectrum antibacterial potency by inhibiting multiple penicillin-binding proteins (PBPs) of both *E. coli* and *S. aureus*. As shown in Fig. 1c and d, under the co-treatment conditions, only *E. coli* showed a significant growth defect during three hours of drug treatment while *S. aureus* remained at normal growth. We found that *Bacillus subtilis*, a rod-shaped bacterium, showed no detectable change under the same condition (Fig. S2). Although we used an MRP susceptible *E. coli* strain, we found a difference between cotreatment (PMBN +MRP) and MRP only treatment. These data suggest that PMBN exerts its action through the recognition of LPS, and this property confers potency against Gram-negative bacteria in co-culture conditions. This is consistent with previous observations (30, 31).

### PMBN leads to improved eradication of persister cells during antibiotic treatment

Persister cells have been thought of as an important subpopulation that evolves into resistant mutants when bacteria are exposed to antibiotics (32). Since PMBN drives the efficient penetration of antibiotics in Gram-negative bacteria, we reasoned that PMBN could potentiate persister killing by antibiotics. To evaluate this, we examined three Gram-negative bacteria including clinical isolates: *A. baumannii, K. pneumoniae*, and *E. coli*. First, to exclude the possibility of pre-existing antibiotic resistance in the clinical isolates, we determined the MICs of a panel of antibiotics. As shown in Fig. S1, all isolates showed no resistance against the tested antibiotics.

**Fig 2:**
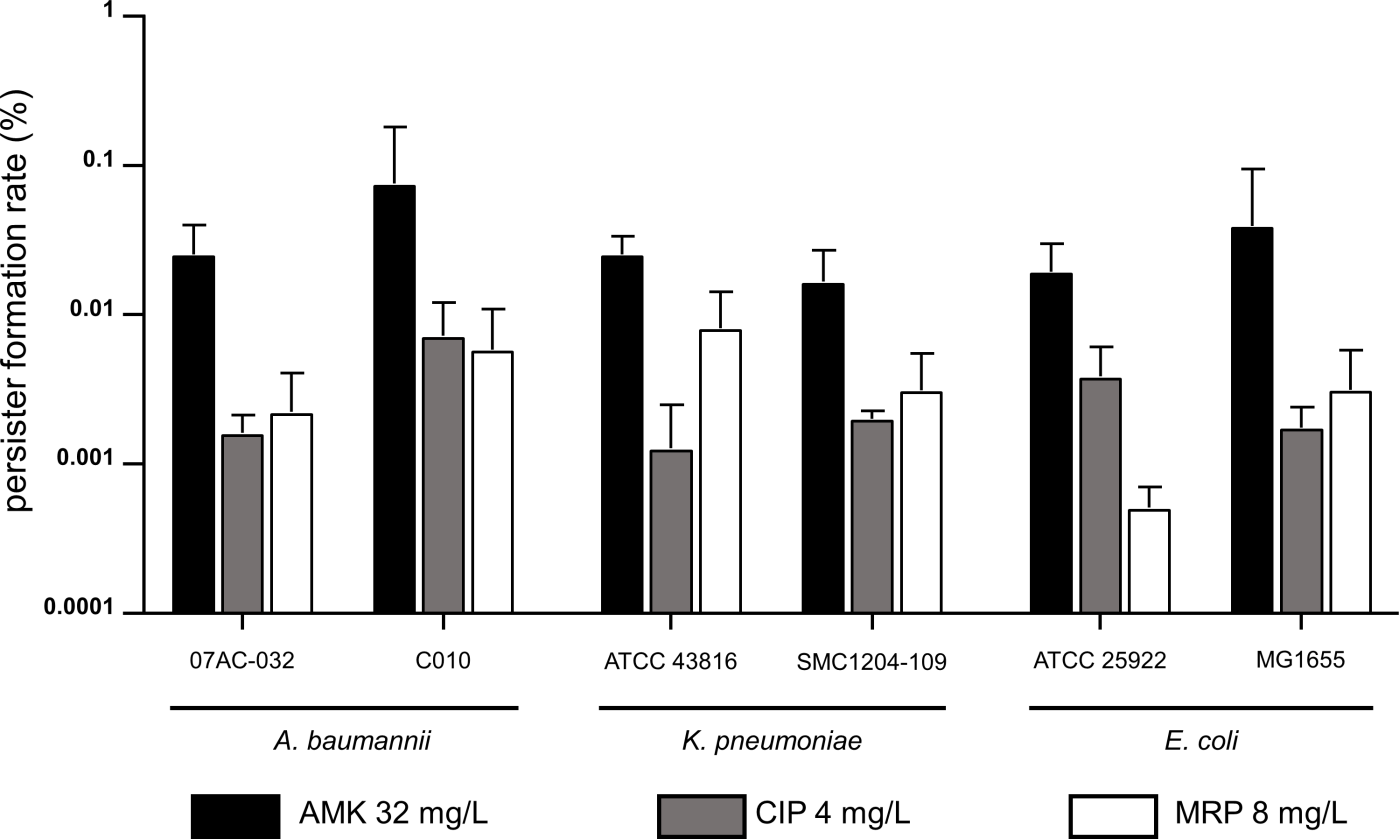
Persister formation of Gram-negative bacteria in the presence of antibiotics was measured. Three antibiotics from different mechanisms were examined to measure the persister formation: amikacin, ciprofloxacin, and meropenem against *A. baumannii, K. pneumoniae,* and *E. coli*. The average and standard deviation for each group were from biological triplicates. *; *P* ≤ 0.05, **; *P* ≤ 0.01.

For the persister assay, we chose three antibiotics based on their cellular targets and penetration mechanism: amikacin is an aminoglycoside with an intracellular target and is dependent on the inner membrane potential to penetrate the cell, ciprofloxacin can cross the cellular membrane by passive transport, and meropenem is a beta-lactam which requires outer membrane penetration. As shown in Fig. S3, we observed a typical biphasic killing curve during the treatment of these antibiotics, validating our conditions for the formation of the persister subpopulation. The elbow points of the plot were observed after 4-hour antimicrobial exposure. Thus, we chose the subpopulation surviving after 6 hours of antibiotic challenge as persister cells (33, 34). We measured persister formation after treatment with the chosen antibiotics: amikacin, meropenem, ciprofloxacin (Fig. 2), and we were able to collect persisters ranging in the CFU count of 10^−6^ to 10^−3^, which was comparable to previously reported results (35–37).

**Fig 3:**
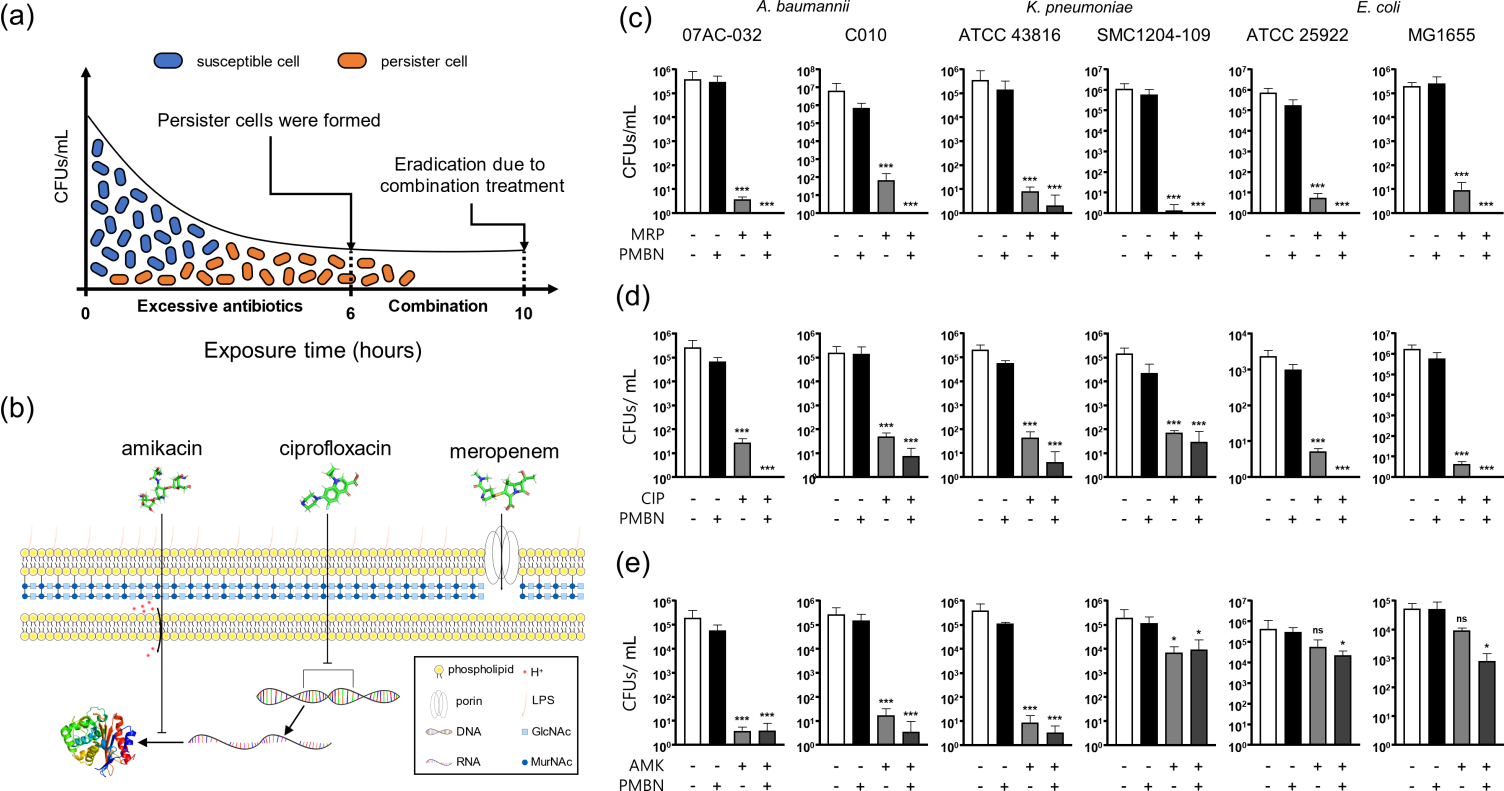
Co-treatment of PMBN with antibiotics decreased and eliminated persisters in three distinct species. (a) Scheme of persister induction performed in this study and (b) scheme depicting mode of action of the three antibiotics used. Persister cells were selected by excessive antibiotic treatment and eliminated by co-treatment of PMBN (2 mg/L). The three antibiotics used in this study have different mode of actions: amikacin, ciprofloxacin, and meropenem. (c) Co-treatment of meropenem (MRP, 8 mg/L) and PMBN was most effective in eliminating persisters. (d) Co-treatment of ciprofloxacin (CIP, 4 mg/L) and PMBN eliminated *E. coli* persisters effectively. (e) Co-treatment of amikacin (AMK, 32 mg/L) and PMBN lead to decrease the persisters of *A. baumannii* and *K. pneumoniae*. *; *P* ≤ 0.05, ***; *P* ≤ 0.001.

We then treated the bacterial strains for 10 hours with the three antibiotics in the presence of PMBN and determined the eradication of persister cells (Fig. 3a and b). Overall, co-treatment with PMBN was significantly more effective in killing persisters of the tested strains compared to antibiotic-only treatment. Hypermucoviscous *K. pneumoniae* ATCC 43816 appeared less responsive to PMBN, suggesting that possible modifications of the bacterial envelope might inhibit the action of PMBN. As expected, potentiation by PMBN varied depending on the location of the cellular target of the antibiotics. In the case of meropenem, which targets peptidoglycan in the periplasmic space, treatment with 2 mg/L PMBN showed a dramatic decrease in persisters compared to meropenem-only treatment, suggesting that PMBN interacts primarily with the outer membrane of Gram-negative bacteria (Fig. 3c). Similarly, when we treated with ciprofloxacin and PMBN, we observed an improved eradication of persister cells, although less potent compared to meropenem (Fig. 3d) (38). This may be because the cellular target of ciprofloxacin is DNA gyrase which is located in the cytoplasmic space; therefore, any changes in the inner membrane during co-treatment may result in a decrease of the potency of PMBN. In the case of amikacin, which targets 30S ribosome in the cytoplasmic space, co-treatment with PMBN at 2 mg/L showed only marginal potentiation (Fig. 3e). This may be because amikacin is imported by an active transport system requiring ATP consumption that is less active in persister cells (39, 40). The increase in the potentiation by addition of PMBN was measured to be significantly higher compared to colistin since PMBN itself is not antibacterial at the given concentration (Fig. 1b). Interestingly, we observed a relatively lower potency against three strains: *E. coli* ATCC 25922, *E. coli* MG1655, and *K. pneumonia* SMC1204-109, with amikacin, even though these strains appeared to be equally responsive under the conditions of ciprofloxacin and meropenem. As previously reported (41–43), in Gram-negative bacteria, the dormant state and resuscitation of persister are related to outer membrane porin proteins such as OmpA which interacted with the receptor of cAMP. The compromised outer membrane by PMBN may hinder porins proteins including OmpA, which can lead to kill persister cells. However, more genetic and mechanistic analyses of the tested strains remain to be studied. We chose these three bactericidal antibiotics because the phenotype of persisters becomes more measurable with them (22).

**Fig 4:**
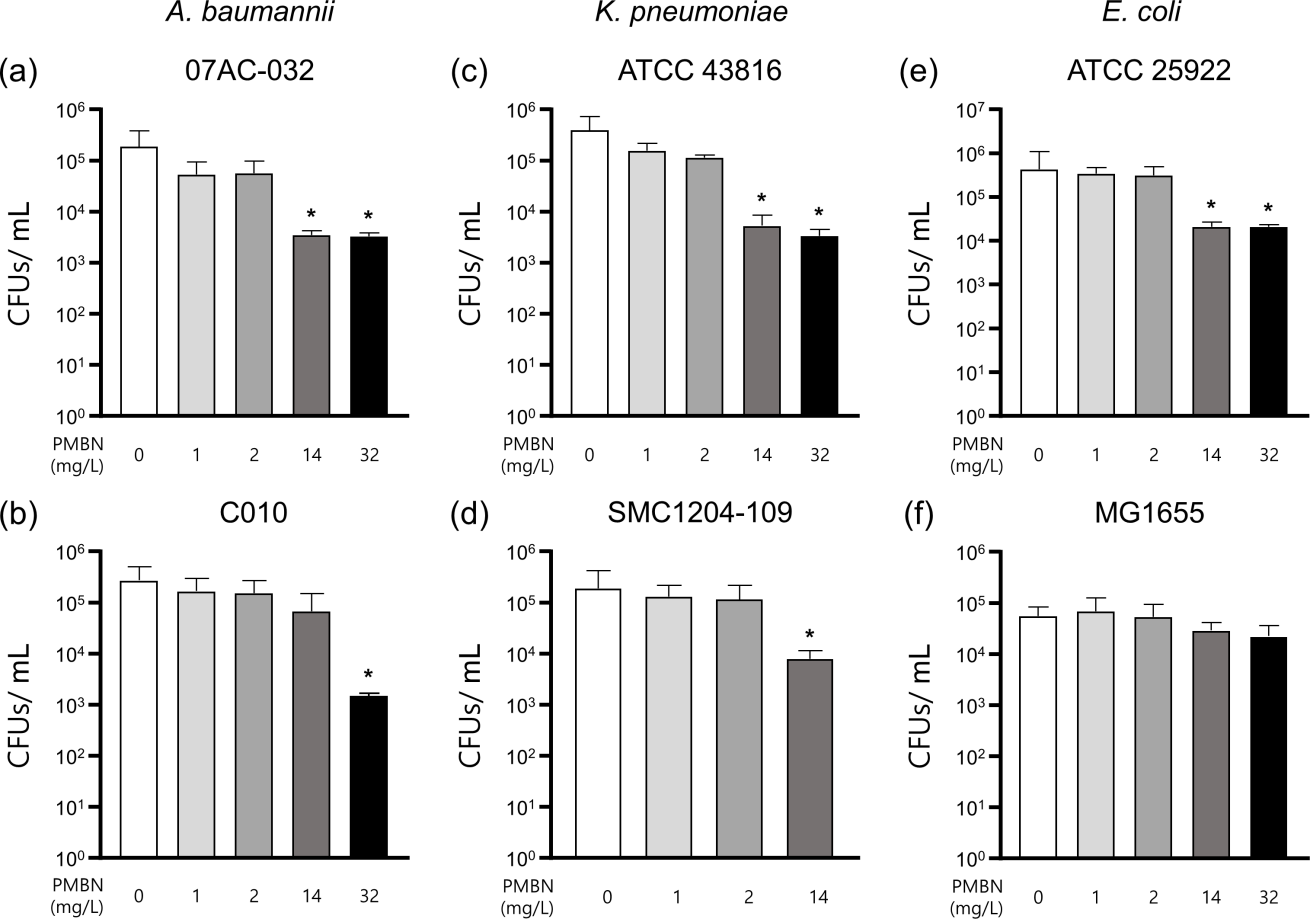
PMBN alone reduced the formation of persister cells. Gram-negative bacteria were treated with PMBN alone for 4 hours at a series of concentration (1–32 mg/L) after persister formation using 32 mg/L AMK. (**a and **b) *A. baumannii*, (**c and **d) *K. pneumoniae*, and (**e and **f) *E. coli* was tested. The average and standard deviation for each group were from biological triplicates.

Subsequently, we speculated that PMBN alone could decrease the occurrence of persister formation. Indeed, as shown in Fig. 4, when we tested using various concentrations of PMBN (1–32 mg/L) which exert no observable impact on the growth, interestingly, the formation of persisters decreased in a dose-dependent manner except for *E. coli* MG1655. It has been suggested that reactive oxygen species (ROS) may be involved during polymyxin treatment. However, unlike other polymyxins, PMBN, which lacks antimicrobial activity, may not induce ROS, as it lacks the 6-methyl group required for the penetration and disruption of the outer membrane (6). Therefore, we note here that since the cytotoxicity of PMBN is much lower than other polymyxins such as colistin, a much higher dose could be used in clinic to eradicate the persister formation (44).

### PMBN suppresses the acquisition of antibiotic resistance in bacterial cell

**Fig 5:**
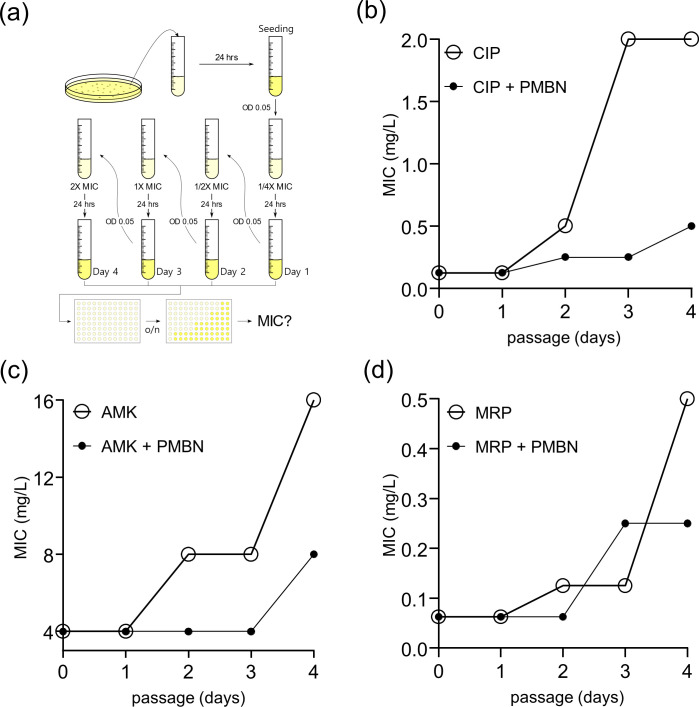
PMBN co-treatment suppressed acquisition of antibiotic resistance. (a) Schematic of the induction of *in vitro* resistance against antibiotics using *E. coli* MG1655. Every 24 hours cultures were dilated to OD_600_ = 0.05 in fresh media containing higher concentration of antibiotics. The MIC of four selected generations (0.25 × MIC, 0.5 × MIC, 1 × MIC, and 2 × MIC) was examined. (b, c, and d) the changes of MIC against each antibiotic in the presence of PMBM were monitored: (b) CIP, (c) AMK, and (d) MRP. The result is a representative of three biological replicates.

Next, we sought to test whether co-treatment with PMBN suppresses the formation of antibiotic-resistant strains. To this end, we performed an assay to raise spontaneous resistant mutants of *E. coli* under serial exposure to increased concentrations of antibiotics (amikacin, ciprofloxacin, and meropenem) in the presence of PMBN (Fig. 5a). For this experiment, taking advantage of the PMBN’s low toxicity profile, we used a high dose of PMBN, 8 mg/L. As shown in Fig. 5b to d, we found that despite PMBN itself having no antibacterial potency, the addition of PMBN significantly lowered the frequency of resistance compared to antibiotic-only treatment for all three antibiotics, which is consistent with our results of PMBN potentiation under co-treatment. Interestingly, among the three tested antibiotics, co-treatment with ciprofloxacin most significantly reduced the formation of resistant mutants (8-fold lower at 1 x MIC: 0.25 mg/L → 2 mg/L) (Fig. 5b). In the case of amikacin co-treatment, a similar increase in the eradication of the resistant population (2-fold lower at 1 x MIC: 4 mg/L → 8 mg/L) was found (Fig. 5c). Interestingly, we found that the meropenem cotreatment was inconclusive in supressing the antibiotic resistance (Fig. 5d), although the cotreatment led to a dramatic eradication of the resistant population as shown in Fig. 3c. This may be due to the relatively higher expression of outer membrane pumps including Omp1 (45). In addition to the eradication of persisters, our data emphasise the utility of co-treatment with PMBN as a strategy to reduce the frequency of the formation of possible antibiotic-resistant mutants.

### Conclusion

We demonstrated that PMBN could potentiate antibiotics selectively in Gram-negative bacteria. Furthermore, since PMBN targets the outer membrane without the lethal effect of its parent compound polymyxin B, a higher dose PMBN can be used. Importantly, this property of PMBN can be applicable to treat Gram-negative bacterial infections with minimal perturbation of beneficial Gram-positive bacteria of the normal microbiota. In addition, we demonstrated that co-treatment with PMBN killed persister and resistant cells more effectively, and also that the treatment of PMBN alone reduced the formation of persister cells. Since our studies showed strong *in vitro* potentiation of the non-toxic and highly selective compound PMBN, further demonstration using *in vivo* infection may be needed. The current form of PMBN can also be modified and improved to have better potentiation on antibiotics and selectivity, and this can lead to the development of novel therapeutic strategies against Gram-negative bacterial infections in the clinic.

## SUPPLEMENTAL MATERIAL

Supplemental figures spectrum.03687-23-s0001.pdf Fig. S1 to S3.

ASM does not own the copyrights to Supplemental Material that may be linked to, or accessed through, an article. The authors have granted ASM a non-exclusive, world-wide license to publish the Supplemental Material files. Please contact the corresponding author directly for reuse.
