# Supplementary material for: Polymyxin B nonapeptide potentiates the eradication of Gram-negative bacterial persisters
Source: Microbiol Spectr. 2024 Feb 23;12(4):e03687-23. doi: 10.1128/spectrum.03687-23 (PMC10986493; doi:10.1128/spectrum.03687-23)
Supplement: Supplemental figures — Fig. S1 to S3. [file spectrum.03687-23-s0001.pdf]

Supplementary Information

Polymyxin B nonapeptide potentiates the eradication of Gram-negative bacterial persisters

Sun Ju Kim[1,†], Jeongwoo Jo[2,†], Jihyeon Kim[1], Kwan Soo Ko[2,*], Wonsik Lee[1,*]

[1] School of Pharmacy, Sungkyunkwan University, Suwon 16419, Republic of Korea
[2] Department of Microbiology, School of Medicine, Sungkyunkwan University, Suwon 16419, Republic of Korea.

[*]Corresponding authors:

Wonsik Lee (wonsik.lee@skku.edu) and Kwan Soo Ko (ksko@skku.edu)

[†]These authors contributed equally to this work

Bacterial species	strains	MICs (mg/L)						
		AMK	CIP	CST	CTX	MRP	PMBN	TET
Acinetobacter baumannii	07AC-032	4	0.125	0.25	2	0.125	≥ 64	0.5
	C010	4	0.125	0.25	2	0.125	≥ 64	0.25
Klebsiella pneumoniae	ATCC 43816	2	≤ 0.06	2	≤ 0.06	≤ 0.06	≥ 64	1
	SMC 1204-109	2	≤ 0.06	0.25	0.125	≤ 0.06	≥ 64	2
Escherichia coli	ATCC 25922	4	≤ 0.06	2	≤ 0.06	≤ 0.06	≥ 64	1
	MG1655	4	≤ 0.06	1	≤ 0.06	≤ 0.06	≥ 64	0.5
Staphylococcus aureus	HG003	4	0.125	≥ 64	1	≤ 0.06	≥ 64	0.5

Supplementary Figure 1. All isolates used in this study had no resistance against 6 antimicrobials tested, while PMBN showing no antimicrobial activity against them. The *K. pneumoniae* isolates are both hypermucoviscous. AMK; amikacin, CIP; ciprofloxacin, CST; colistin, CTX; cefotaxime, MRP; meropenem, PMBN; polymyxin B nonapeptide, TET; tetracycline.

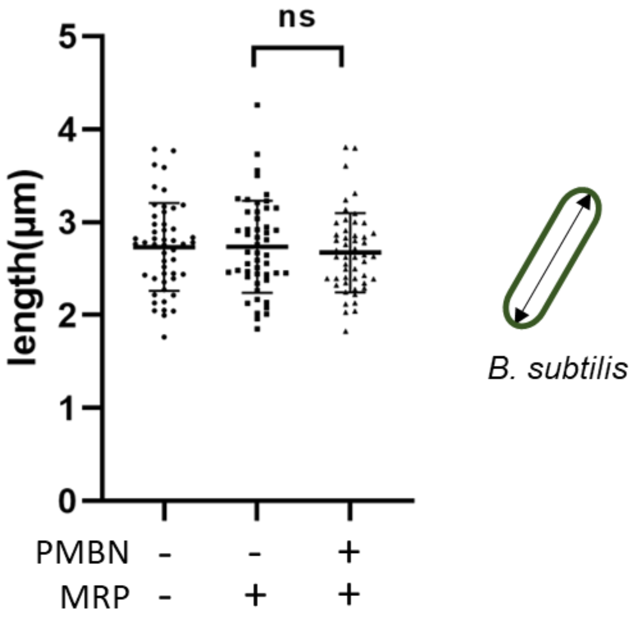

Supplementary Figure 2. Co-treatment of Polymyxin B nonapeptide (PMBN) and meropenem at sub MIC has no effect on *Bacillus subtills.* *B. subtilis* BY79 was treated with meropenem (MRP) only or in the presence of 1 mg/L PMBN for three hours. The cell width was measured using ImageJ (version 1.54d). The lengths of a total of 50 cells were measured. The difference between MRP alone and cotreatment is not statistically significant.

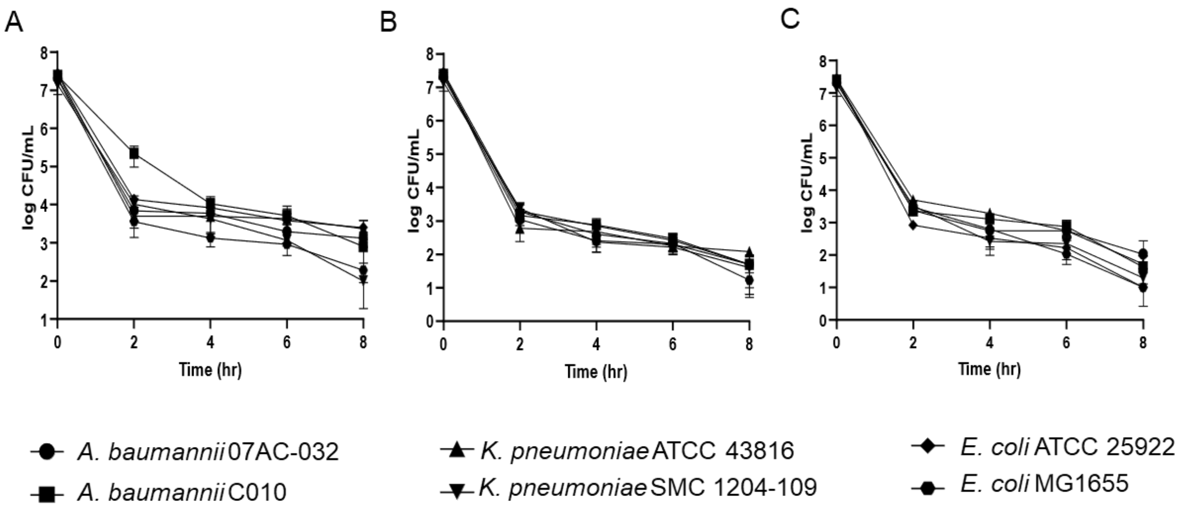

Supplementary Figure 3. Survived subpopulation during antimicrobial exposure was examined. All isolates of Gram-negative bacteria showed biphasic time-kill curves against all three antimicrobials evaluated: amikacin **(A)**, ciprofloxacin **(B)**, and meropenem **(C)**. Also, none of them showed regrowth from acquired resistance by single overnight exposure to the antimicrobials. The average and standard deviation are drawn by the result of triplicate repeats.
